# Predicting the efficacy of radiotherapy for esophageal squamous cell carcinoma based on enhanced computed tomography radiomics and combined models

**DOI:** 10.3389/fonc.2023.1089365

**Published:** 2023-03-16

**Authors:** Jihui Liu, Xiyue Yang, Xin Mao, Tingting Wang, Xuhai Zheng, Gang Feng, Tangzhi Dai, Xiaobo Du

**Affiliations:** Department of Oncology, National Health Commission (NHC) Key Laboratory of Nuclear Technology Medical Transformation (Mianyang Central Hospital), Mianyang Central Hospital, School of Medicine, University of Electronic Science and Technology, Mianyang, China

**Keywords:** radiotherapy, esophageal squamous cell carcinoma, computed tomography, radiomics, dosimetric

## Abstract

**Purpose:**

This study aimed to investigate the ability of enhanced computed tomography (CT)-based radiomics and dosimetric parameters in predicting response to radiotherapy for esophageal cancer.

**Methods:**

A retrospective analysis of 147 patients diagnosed with esophageal cancer was performed, and the patients were divided into a training group (104 patients) and a validation group (43 patients). In total, 851 radiomics features were extracted from the primary lesions for analysis. Maximum correlation minimum redundancy and minimum least absolute shrinkage and selection operator were utilized for feature screening of radiomics features, and logistic regression was applied to construct a radiotherapy radiomics model for esophageal cancer. Finally, univariate and multivariate parameters were used to identify significant clinical and dosimetric characteristics for constructing combination models. The area evaluated the predictive performance under the receiver operating characteristics (AUC) curve and the accuracy, sensitivity, and specificity of the training and validation cohorts.

**Results:**

Univariate logistic regression analysis revealed statistically significant differences in clinical parameters of sex (p=0.031) and esophageal cancer thickness (p=0.028) on treatment response, whereas dosimetric parameters did not differ significantly in response to treatment. The combined model demonstrated improved discrimination between the training and validation groups, with AUCs of 0.78 (95% confidence interval [CI], 0.69–0.87) and 0.79 (95% CI, 0.65–0.93) in the training and validation groups, respectively.

**Conclusion:**

The combined model has potential application value in predicting the treatment response of patients with esophageal cancer after radiotherapy.

## 1 Introduction

Esophageal cancer is the eighth most prevalent and sixth most lethal cancer worldwide. In Asia and Eastern Europe, the most prevalent histological subtype of this malignancy is squamous cell carcinoma (1). More than 70% of patients with esophageal cancer are diagnosed at an intermediate to advanced stage, with unresectable or metastatic disease, and a combination of chemotherapy and radiation therapy is frequently provided to patients with esophageal cancer (2). Studies have indicated that the 5-year survival rate for patients with locally advanced esophageal cancer treated with radiation is only 36–47% (3, 4), and the 5-year overall survival of patients with complete remission (CR) is better than that of patients without CR (5). Therefore, early identification of patients who do not respond to radiotherapy and prompt monitoring of tumor response to treatment during radiotherapy are crucial for implementing individualized precision radiotherapy and enhancing overall patient survival.

Computed tomography (CT) is commonly used to assess the preoperative staging of esophageal cancer, including the extent of infiltration, lymph node extent, and metastasis, for clinical treatment decisions (6). However, CT only shows the external morphological features of esophageal cancer. It is challenging to fully assess the heterogeneity within the tumor. Radiomics extracts quantitative CT image features with a high throughput. This information extraction is based on the entire tumor and is not confined to a single tissue sample, allowing for a thorough description of tumor heterogeneity. Hou et al. investigated the baseline CT-enhanced image characteristics of 49 patients (33 with strong response and 16 with poor response) with esophageal cancer treated with radiation and found substantial differences in kurtosis and skewness in histogram characteristics between the two groups (7). Yang et al.’s analysis of patients receiving lower doses of neoadjuvant chemoradiotherapy (nCRT) did not reveal any clinical characteristics that predicted patients’ arrival to pathological complete response (pCR). However, radiomics features enabled the construction of three highly accurate models for predicting pCR following nCRT in individuals with esophageal cancer (8). Some researchers have attempted to predict an outcome by combining intratumoral and peritumoral features. Radiomics examination is not restricted to the tumor body. Hu et al. included patients with esophageal cancer who underwent surgery after nCRT in two institutions and extracted radiomics features from baseline-enhanced CT intratumoral and peritumoral regions to construct models, demonstrating that models constructed with seven intratumoral and six peritumoral radiomics features had superior predictive performance, with receiver operating characteristic (ROC) curves of 0.906 and 0.85 in the training and validation groups, respectively (9).

With the progress of radiotherapy technology, esophageal cancer can be treated by three-dimensional conformal radiation therapy (3D-CRT), intensity-modulated radiotherapy (IMRT), and volumetric-modulated arc therapy (VMAT), but its 5-year survival rate remains inadequate. Local uncontrolled or recurrence remains the most common cause of radiotherapy failure. Due to individual variances, the radiation dose for each patient varies. Some studies have demonstrated the significant efficacy of radiotherapy up to a dose of 40 Gy in certain patients, whereas others are not sensitive to radiotherapy and fail to improve their local control rate even when administered 70 Gy (10). Incremental radiation therapy dosages may result in severe toxic side effects, the severity of which is mostly determined by clinical criteria and the quantity of healthy tissue surrounding the exposed tumor. In radiation therapy for cancer, metrics, such as prescribed dose, dose distribution, and dose-volume histogram, can also be utilized to evaluate treatment response and cancer prognostic analysis (11, 12).

To the best of our knowledge, the doses of radiotherapy received by patients in some current studies were also significantly lower than those of radical radiotherapy. Few studies have incorporated dosimetric data and several other variables into predictive models. To assist clinicians in deciding the best course of treatment for patients with esophageal cancer receiving radiation, this study aimed to examine the effects of enhanced CT-based radiomics in predicting the response to radiotherapy.

## 2 Materials and methods

### 2.1 Patients and treatment

The ethical committee allowed a retrospective collection of 147 patients with a histological diagnosis of esophageal squamous cell carcinoma at our hospital between January 2018 and December 2021 (approval number: S2022035-01). The inclusion criteria were as follows: (a) patients with a histopathology-confirmed squamous cell carcinoma of the esophagus, (b) patients who had completed radiotherapy, (c) patients without distant metastases or other neoplastic diseases, and (d) patients with trackable treatment results. The exclusion criteria were as follows: (a) patients with missing follow-up data; (b) patients who had previously undergone chest radiation, chemotherapy, or surgical tumor excision; (c) patients with multifocal primary disease; and (d) extreme respiratory motion artifacts; and (e) invisible tumor on CT image. Image quality is judged by the two radiologists independently, and the disagreement is resolved through negotiation. Patients underwent 3D-CRT, IMRT, or VMAT during the treatment period. In total, 100% of the prescribed dose encompassed 95% of the volume of the target area for all patients.

### 2.2 Response assessment

After 3 months of treatment, response to treatment was assessed by CT findings and determined according to the efficacy evaluation criteria for solid tumors (Response Evaluation Criteria in Solid Tumors) (13). CR, partial response (PR), stable disease (SD), and progressive disease (PD) were assessed. Patients with CR or PR were classified as responders, whereas patients with SD or PD were classified as nonresponders.

### 2.3 Image acquisition

All patients underwent chest CT examinations utilizing a Siemens large-aperture CT scanner. The scan parameters (tube voltage, 120 kVp; tube current, 200 mAs; matrix, 512×512; layer thickness, 5 mm; layer spacing, 5 mm) were in accordance with the clinical standard acquisition methodology. Iodine contrast agent was injected at 3 ml/s using a high-pressure syringe. A radiation oncologist drew the primary gross tumor volume (GTV) on Oncentra software, which was subsequently examined by an experienced radiation oncologist. Avoiding the esophagus lumen, blood arteries, periesophageal fat, and artifacts were outlined as the GTV.

### 2.4 Feature extraction

TPS exported Digital Imaging and Communications in Medicine files to 3D Slicer(version,4.11, https://www.slicer.org) for preprocessing (1×1×1 resampling) and feature extraction (**Supplementary Figure 1**) (14). In total, 851 features, comprising 107 original features and 744 wavelet features, were extracted from each GTV. The original features included 18 first-order statistical features, 14 shape size features, 14 gray-level dependence matrix, 16 gray-level size zone matrix, 24 gray-level co-occurrence matrix,16 gray-level run-length matrix, and 5 neighboring gray tone difference matrix. Image transformation features, such as wavelet transform features, were primarily utilized to divide original tumor images into distinct frequency domains. Except for 14 shape features that do not change with image transformation, each of the 93 features is extracted to different values in the image GTV after 8 wavelet transforms.

### 2.5 Feature screening and model construction

Random stratified sampling was used to divide 147 patients into two groups (104 and 43 patients in the training and validation groups, respectively). Data standardization and feature extraction were performed using R software (version 3.6.0, https://www.r-project.org).The extracted features were preprocessed with Z-score for normalization to reduce the effect of different magnitudes on the features, specifically by eliminating the mean of each feature to center the feature values and then dividing by the standard deviation of each feature. The minimum redundancy maximum relevance (mRMR) algorithm was then used to screen features. The mRMR algorithm is based on calculating a pair of correlation coefficient (A) and redundancy coefficient value (B) for each feature, where the correlation coefficient represents the relationship between the feature and treatment response and the redundancy coefficient represents the redundancy coefficient between features. The A-B values of all parameter values for features were then ordered in decreasing order (15). The least absolute shrinkage and selection operator (LASSO) method was then utilized for additional feature screening using tenfold cross-validation. A logistic regression model calculated a radiomics score (Rad-score) for each patient using model-weighted coefficients.

### 2.6 Model construction and evaluation

We established a combined model to predict the efficacy of radiotherapy for esophageal cancer by using multivariate logistic regression analysis. The variables included Rad-score, clinical and dosimetric parameters. The Combine model is finally demonstrated through a nomogram. The performance of the model was evaluated using area under the curve (AUC), precision, sensitivity, and specificity. Using a decision curve analysis, the quantification of net benefits under different threshold probabilities was confirmed.

### 2.7 Statistical analyses

All statistical analyses were performed using the R software. Continuous variables are expressed as median (Q1, Q3) using the Mann–Whitney U test. For the count data, the Fisher’s exact probability approach was utilized. Univariate and multivariate logistic regression analyses were performed to identify the independent predictors of clinical and dosimetric indicators. The difference in AUC between models was examined using the Delong test. *P*<0.05 was considered statistically significant.

## 3 Results

In total, 236 patients with esophageal cancer were treated in our hospital, of whom 15 discontinued treatment, 23 were lost to follow-up, 22 had a history of radiotherapy, 10 had incomplete data and19 had poor image quality. These patients were excluded from the statistical analyses. The remaining 147 patients (113 males and 34 females; median age, 66 years) met the inclusion criteria. The number of patients who responded to treatment (CR+PR) was 89, whereas 58 patients (PD+SD) were nonresponders. The clinical and dosimetric characteristics of the patients are shown in **Table 1**.

**Table 1:** The clinical and dosimetric characteristics of the patients.

Characteristic	Non-response (n=58)	Response (n=89)	*p*
Age	67.00 (59.00,73.00)	66.00 (60.00,73.00)	0.899
Gender			0.008*
Female	20 (34.48%)	14 (15.73%)	
Male	38 (65.52%)	75 (84.27%)	
Tumor location			0.176
Cervical	5 (8.62%)	2 (2.25%)	
Upper	12 (20.69%)	17 (19.10%)	
Middle	27 (46.55%)	54 (60.67%)	
Lower	14 (24.14%)	16 (17.98%)	
Histologic grade			0.141
Poor	24 (41.38%)	24 (26.97%)	
Moderate	31 (53.45%)	62 (69.66%)	
Well	3 (5.17%)	3 (3.37%)	
T stage			0.812
T1	2 (3.45%)	1 (1.12%)	
T2	11 (18.97%)	18 (20.22%)	
T3	30 (51.72%)	44 (49.44%)	
T4	15 (25.86%)	26 (29.21%)	
N stage			0.373
N0	14 (24.14%)	14 (15.73%)	
N1	30 (51.72%)	43 (48.31%)	
N2	13 (22.41%)	30 (33.71%)	
N3	1 (1.72%)	2 (2.25%)	
M stage			0.765
M0	54 (93.10%)	83 (93.26%)	
M1	4 (6.90%)	6 (6.74%)	
Group stage			0.309
I	2 (3.45%)	0 (0.00%)	
II	13 (22.41%)	19 (21.35%)	
III	26 (44.83%)	38 (42.70%)	
IV	17 (29.31%)	32 (35.96%)	
Hypertension			0.865
Yes	6 (10.34%)	10 (11.24%)	
No	52 (89.66%)	79 (88.76%)	
Smoking history			0.466
Yes	15 (25.86%)	28 (31.46%)	
No	43 (74.14%)	61 (68.54%)	
Drinking history			0.748
Yes	13 (22.41%)	22 (24.72%)	
No	45 (77.59%)	67 (75.28%)	
Nutrition			0.154
1	18 (31.03%)	35 (39.33%)	
2	17 (29.31%)	27 (30.34%)	
3	6 (10.34%)	13 (14.61%)	
4	13 (22.41%)	7 (7.87%)	
5	4 (6.90%)	7 (7.87%)	
Thickness	1.35 (1.19,1.60)	1.50 (1.17,1.90)	0.048*
Length	5.50 (4.50,7.00)	5.90 (4.50,7.00)	0.959
BMI	22.00 (19.59,23.42)	21.50 (19.80,23.30)	0.834
Dose	60.00 (60.00,60.00)	60.00 (60.00,60.00)	0.650
Frequency	30.00 (28.00,30.00)	30.00 (28.70,30.00)	0.718
Divided dose	2.00 (2.00,2.13)	2.00 (2.00,2.00)	0.352
PTV			
Dmin (Gy)	5400.00 (4952.10,5601.20)	5306.00 (4786.50,5596.50)	0.660
Dmax (Gy)	6636.00 (6518.50,6722.50)	6599.00 (6478.90,6757.90)	0.641
Dmean (Gy)	6252.25 (6203.85,6293.10)	6228.00 (6167.00,6286.60)	0.374
V90 (%)	99.99 (99.86, 100.00)	99.99 (99.86, 100.00)	0.815
V93 (%)	99.78 (99.45, 99.99)	99.86 (99.44, 99.99)	0.837
V95 (%)	99.43 (99.14, 99.71)	99.57 (98.99, 99.87)	0.576
Lung			
Dmean (Gy)	1169.00 (1047.85,1366.30)	1200.00 (1007.10,1341.30)	0.984
V5 (%)	53.52 (48.52, 58.63)	51.72 (44.51, 58.68)	0.429
V10 (%)	37.41 (34.60, 41.99)	38.51 (33.31, 41.38)	0.967
V20 (%)	21.41 (18.81, 27.59)	23.04 (19.82, 26.05)	0.898
V30 (%)	11.49 (8.04, 14.55)	12.45 (8.22, 14.82)	0.756
V40 (%)	5.68 (3.88, 8.34)	5.94 (3.52, 8.37)	0.997
Heart			
Dmean (Gy)	2748.00 (1640.10,3255.50)	2598.00 (1108.20,3190.70)	0.234
V5 (%)	92.89 (57.94, 98.43)	87.07 (38.63, 97.13)	0.134
V10 (%)	79.00 (48.04, 91.82)	75.95 (32.61, 89.80)	0.242
V15 (%)	69.00 (41.20, 83.75)	62.35 (24.00, 80.68)	0.203
V20 (%)	61.52 (36.47, 74.22)	53.89 (19.91, 73.42)	0.241
V25 (%)	54.67 (27.72, 66.24)	46.25 (17.49, 64.02)	0.229
V30 (%)	43.34 (21.91, 55.79)	39.34 (14.13, 50.50)	0.161
V40 (%)	24.34 (12.63, 39.00)	20.31 (7.47, 32.04)	0.210
V50 (%)	10.34 (4.52, 14.97)	7.47 (2.51, 14.51)	0.189
V60 (%)	1.60 (0.00, 4.03)	1.29 (0.00, 3.99)	0.564
Spinal Cord			
Dmax (Gy)	4421.00 (4338.85,4531.00)	4421.00 (4338.60,4521.50)	0.855
Dmean (Gy)	2418.5 (1910.75,2920.75)	2295 (1816.01,2725.60)	0.301

*p<0.05.

**Figure 1:**
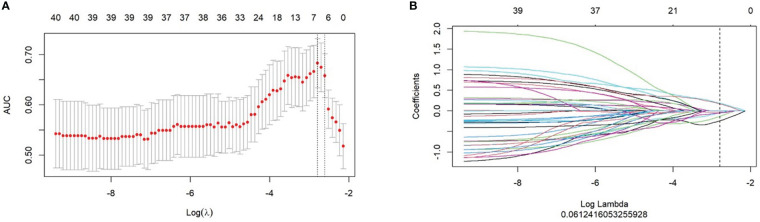
Selection of radiomics features for predicting response using the least absolute shrinkage and selection operator (LASSO) logistic regression model. **(A)** LASSO coefficient profiles of the radiomics features. **(B)** The cross-validation curve.

LASSO regression was used to minimize the dimensionality of the recovered features, and 7 out of 851 possible radiomics features were selected to calculate their products with the regression coefficients using the following equations (**Figures 1A, B**). Each patient’s Rad-score was obtained and calculated as follows: Rad-score=–0.236×Original_firstorder_90Percentile-0.026×Wavelet.Hll_firstorder_Skewness-0.128×Original_glszm_HighGrayLevelZoneEmphasis+0.19×Wavelet.Lhh_glcm_ClusterShade-0.046×Wavelet.Hll_glcm_ClusterShade-0.049×Wavelet.Hll_firstorder_Maximum+0.173×Wavelet.Hhl_glcm_ClusterShade

**Figure 2:**
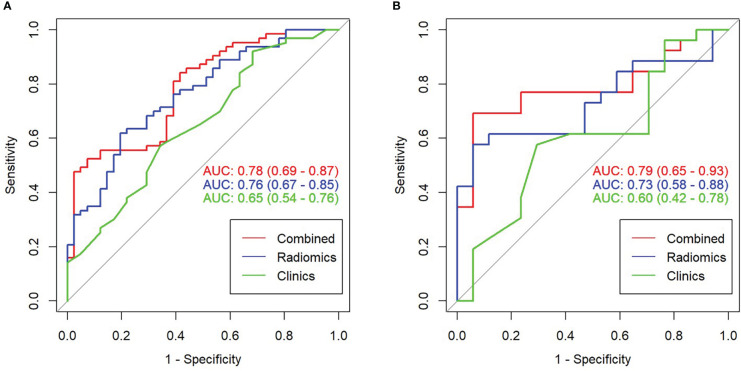
Receiver operating characteristic (ROC) curve comparison of combined and radiomics and clinical models. **(A)** ROC curve in the training set. **(B)** ROC curve in the validation set.

**Figure 2** illustrates the results of using the AUC size of the area under the ROC curve to measure the prediction performance of the model. In the training group, the AUC value of radiomics for predicting esophageal cancer treatment response was 0.76 (95% confidence interval [CI], 0.67–0.85), with an accuracy of 0.692 (95% CI, 0.594–0.779), a sensitivity of 80.5%, and a specificity of 61.9%. In the validation group, the AUC, accuracy, sensitivity, and specificity were 0.73 (95% CI, 0.58–0.88), 0.721 (95% CI, 0.563–0.846), 88.2%, and 61.5%, respectively. The Delong test revealed no statistically significant difference between the effectiveness of the two groups (*p*>0.05).

The clinical and dosimetric parameters related to treatment response in the training group were determined by univariate and multivariate logistic regression analyses. Sex and esophageal carcinoma thickness were substantially associated with treatment response among clinical characteristics, as shown by univariate logistic regression analysis. However, none of the dosimetric variables were related to treatment response (**Table 2**). Hence, sex, esophageal cancer thickness, and Rad-score were incorporated into the multivariate logistic analysis to construct a combined model.

**Table 2:** Univariate and Multivariate logistic regression analysis in the training set.

Variable	Univariate analysis		Multivariate analysis	
	OR (95%CI)	*p*	OR (95%CI)	*p*
Age	0.985 (0.940, 1.032)	0.516		
Gender	2.727 (1.098, 6.771)	0.031*	2.028 (0.705,5.838)	0.189
Tumor location	1.392 (0.823, 2.354)	0.217		
Histologic grade	1.523 (0.707, 3.278)	0.282		
T stage	1.278 (0.726, 2.251)	0.395		
N stage	1.302 (0.759, 2.234)	0.338		
M stage	0.633 (0.121, 3.301)	0.588		
Group stage	1.291 (0.761, 2.189)	0.343		
Treatment	1.599 (0.522, 4.898)	0.411		
Hypertension	1.156 (0.316, 4.229)	0.826		
Smoking history	1.442 (0.593, 3.506)	0.420		
Drinking history	1.404 (0.539, 3.661)	0.487		
Nutrition	0.879 (0.648, 1.191)	0.405		
Medication	1.490 (0.831, 2.674)	0.181		
Thickness	2.419 (1.101, 5.317)	0.028*	2.033 (0.877,4.713)	0.098
Length	0.972 (0.804, 1.176)	0.772		
BMI	1.001 (0.880, 1.139)	0.985		
Dose	0.984 (0.885, 1.094)	0.768		
Frequency	1.027 (0.811, 1.301)	0.824		
Divided dose	0.150 (0.001,21.467)	0.454		
PTV_Dmin	1.000 (1.000, 1.000)	0.576		
PTV_Dmax	1.000 (0.999, 1.000)	0.530		
PTV_Dmean	1.000 (0.999, 1.000)	0.452		
PTV_V90	0.361 (0.039, 3.386)	0.373		
PTV_V93	0.826 (0.294, 2.317)	0.716		
PTV_V95	1.177 (0.553, 2.507)	0.672		
Lung_V5	0.981 (0.943, 1.020)	0.325		
Lung_V10	1.000 (0.946, 1.056)	0.991		
Lung_V20	0.999 (0.931, 1.072)	0.977		
Lung_V30	0.995 (0.975, 1.015)	0.617		
Lung_V40	1.005 (0.970, 1.041)	0.772		
Lung_Dmean	1.000 (0.999, 1.001)	0.979		
Heart_V5	0.992 (0.979, 1.005)	0.236		
Heart_V10	1.001 (0.998, 1.004)	0.572		
Heart_V15	0.993 (0.980, 1.007)	0.325		
Heart_V20	0.993 (0.979, 1.007)	0.334		
Heart_V25	0.993 (0.977, 1.009)	0.368		
Heart_V30	0.990 (0.973, 1.008)	0.265		
Heart_V40	0.992 (0.970, 1.014)	0.465		
Heart_V50	0.985 (0.944, 1.027)	0.469		
Heart_V60	1.023 (0.902, 1.162)	0.720		
Heart_Dmean	1.000 (0.999, 1.000)	0.318		
Spinal_Cord_Dmax	1.001 (0.999, 1.002)	0.292		
Spinal_Cord_Dmean	1.000 (0.999, 1.000)	0.538		
Rad-score	18.861 (4.718,75.403)	<0.01*	15.326 (3.687,63.693)	<0.01*

*p<0.05.

**Figure 3:**
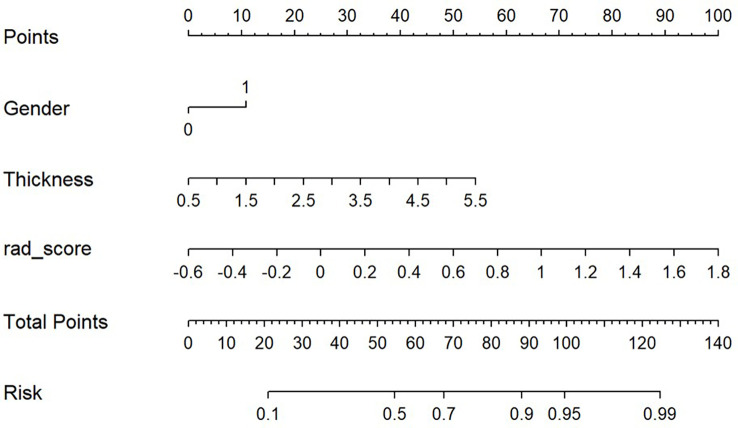
Predictive nomogram combined Rad-score, sex, and esophageal cancer thickness.

Based on the results of the multivariate analysis, a combine model is finally demonstrated through a nomogram (**Figure 3**), The risk ratio and significance of each variable in the multivariate combined model are shown in **Supplementary Table 1**, and the outcomes are presented in **Figure 2**. In the training group, the AUC, accuracy, sensitivity, and specificity for the combined model were 0.78 (95% CI, 0.69–0.87), 0.673 (95% CI, 0.574–0.762), 96.7%, and 54.8%, respectively. In the validation group, the AUC, accuracy, sensitivity, and specificity were 0.79 (95% CI, 0.65–0.93), 0.651 (95% CI, 0.491–0.790), 92.3%, and 53.3%, respectively. The performance metrics of radiomics, clinics, and combined models are displayed in **Table 3**.The AUC of combined model was higher than that of the clinical model, indicating that the combined model achieved considerably better discrimination capability than clinical model(DeLong’s test, *p* < 0.001).However, there was no significant difference between the combined and the radiomics model (*p*=0.772) and between the radiomics and the clinical model (*p*=0.133).

**Table 3:** Predictive performance of radiomics, clinics, and combined models.

Model	Training set (n=104)				Test set (n=43)			
	AUC (95%CI)	Accuracy	Sensitivity	Specificity	AUC (95%CI)	ACC	Sensitivity	Specificity
Radiomics	0.76 (0.67-0.85)	0.692 (0.594-0.779)	80.5%	61.9%	0.73 (0.58-0.88)	0.721 (0.563-0.846)	88.2%	61.5%
Clinics	0.65 (0.54-0.76)	0.683 (0.584-0.771)	67.4%	72.2%	0.60 (0.42-0.78)	0.628 (0.467-0.770)	63.9%	57.1%
Combine	0.78 (0.69-0.87)	0.673 (0.574-0.762)	96.7%	54.8%	0.79 (0.65-0.93)	0.651 (0.491-0.790)	92.3%	53.3%

**Figure 4:**
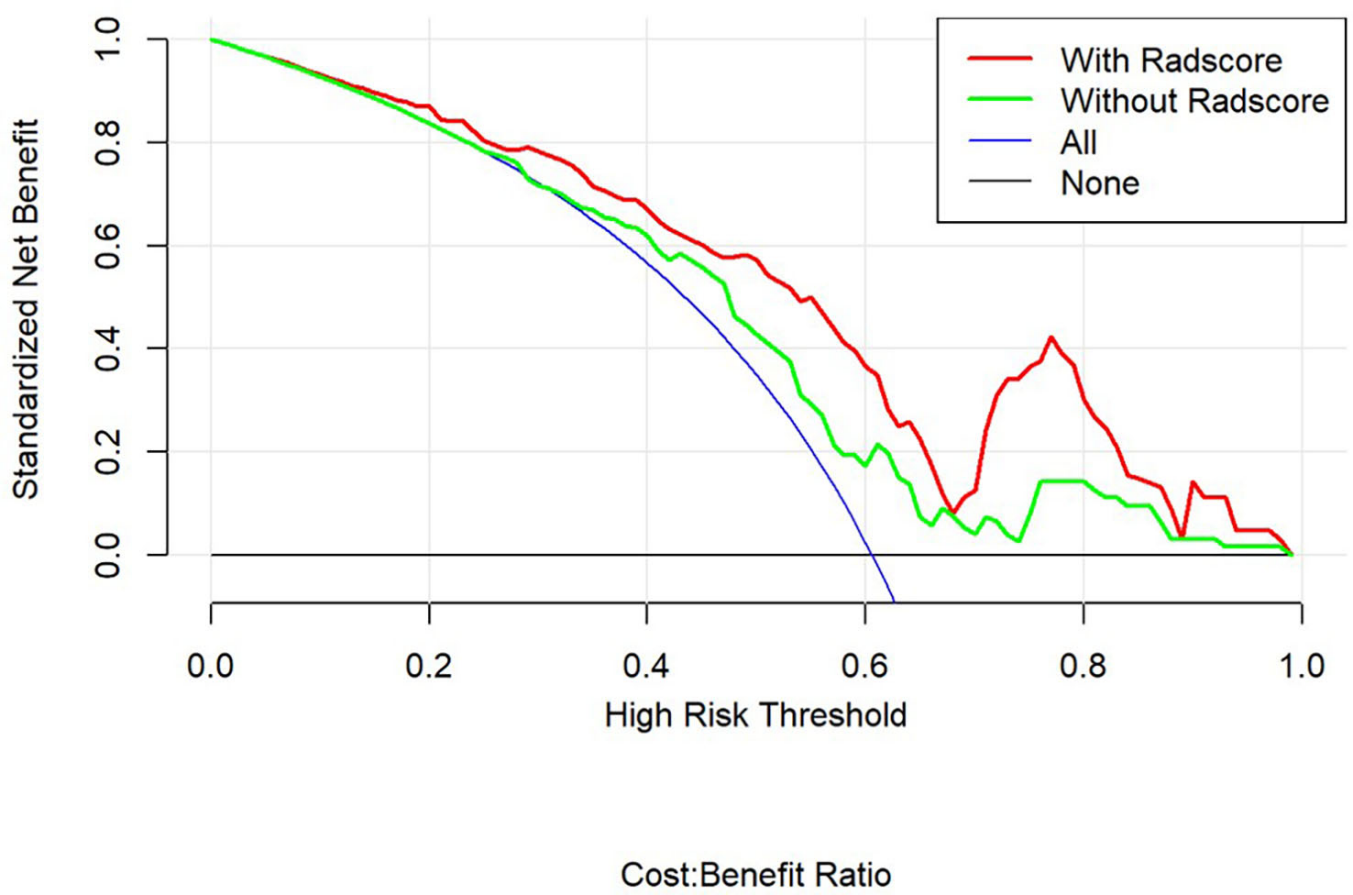
Decision curve analysis for the combined and clinical models.

Using decision curves to analyze the influence of the model on clinical treatment decisions, the clinical model (without Rad-score) or the combined model (with Rad-score) outperformed “all treatment” or “no treatment” when the risk threshold was greater than 10%, and the combined model had greater predictive power than the clinical model when the threshold was more significant than 23% (**Figure 4**).

## 4 Discussion

In this study, radiomics features of localized CT images of patients before radiotherapy were extracted, and the optimal seven features were screened out, combined with clinical features to construct a model of the treatment response of patients receiving radiotherapy, which can provide a cost-effective and noninvasive method for predicting the efficacy of radiotherapy.

In the present study, two clinical factors, esophageal carcinoma thickness and sex, were substantially associated with treatment response. Previous studies have demonstrated the predictive usefulness of esophageal carcinoma thickness in determining preoperative treatment response (16, 17). According to Zhang et al., esophageal cancer thickness as a single predictor can evaluate survival and efficacy of preoperative chemotherapy (18). The limited value of thickness measurement on CT may be attributed to the swelling effect of necrotic and fibrotic tissues following radiation, resulting in persistent imaging abnormalities. Radiomics augments standard imaging parameters. It recognizes intra-tissue heterogeneity, hence increasing the predictive accuracy of the model for tumor response. According to a previous study on esophageal cancer, women are more likely to present with pCR and have higher survival rates than men (19, 20). In contrast, the results of the current study were different, possibly due to the small number of women with esophageal cancer in the study population, which led to unusual experimental results. Dosimetric measures were not altered significantly when the treatment response was reversed. Jin et al. obtained similar results using a dosimetric model to evaluate treatment response in esophageal cancer after radiotherapy (21). The obtained dosimetric parameters may be 3D dose distributions, which describe the volume of irradiation received by an organ at a provided dose. There is a loss of spatial link information between voxels.

Several studies have demonstrated the use of radiomics, an emerging image analysis technique, to predict the efficacy of radiation in patients with esophageal cancer. Murakami et al. retrieved 22 radiomics variables for LASSO regression analysis from positron emission tomography (PET)/CT images of 98 patients with esophageal cancer treated with nCRT. Using a neural network classifier, they developed a prediction model with accuracy, sensitivity, and specificity of 89.6%, 92.7%, and 89.5%, respectively (22). Hou et al. extracted 138 radiomics features from the pre-therapy T2-weighted (T2W)- and spectral attenuated inversion recovery (SPAIR) T2W-magnetic resonance imaging (MRI) sequences of 68 patients with esophageal squamous carcinoma, which could distinguish between CR and stable lesions, partial remission and stable lesions, and reactive and non-reactive lesions by 26, 17, and 33 features, respectively, and used artificial neural networks (ANNs) and support vector machine (SVM) to construct predictive models. The performance of the SPAIR T2W-MRI model was superior to that of the T2W sequence (SVM, 0.929; ANN, 0.883) (23). However, these earlier studies rarely incorporated several elements, such as dosimetric parameters, into model projections. In some of these studies, patients received nCRT, with significantly lower treatment doses than radical radiotherapy. In this study, dosimetric, clinical, and other multiple factors were considered, and the LASSO regression method was used to construct a model for predicting treatment response after radiotherapy in patients with esophageal cancer, with a maximum sensitivity of 96.7% and a maximum AUC of 0.79, indicating that the prediction model has a high level of confidence in identifying treatment response. Yip et al. predicted the treatment response of patients with esophageal cancer based on PET/CT utilizing a radiomics approach. They showed high sensitivity (81%) and specificity (82%) (24), which are comparable to the current study’s findings. Luo et al. studied baseline CT images of 226 patients receiving nCRT for esophageal cancer, and LASSO was used to build Rad-score for seven radiomics features. Combining the radiomics labels with clinical staging, nomograms were created to predict CR, with AUCs of 0.844 and 0.807 for the training and validation groups, respectively. The prediction algorithm based on the nomogram outperformed clinical staging (25). The predictive performance of the combined model was similarly superior to that of the only radiomics model in this study.

Currently, CT-based radiomics characteristics consist primarily of geometric, morphological, textural, and intensity-based histogram characteristics. Textural characteristics are a standard way to assess tumor heterogeneity (26). Yip et al. studied PET/CT images of 31 patients with esophageal cancer before and after nCRT and reported that the grayscale histogram standard deviation (histogram SD) characteristics of tumors before and after therapy were related to tumor regression grade (27). In addition, another study conducted by Yip et al. extracted radiomics features that responded to patient heterogeneity in CT radiomics before and after radiotherapy, such as entropy, homogeneity, mean gray intensity, kurtosis, and standard deviation of the histogram. After comparing the changes in these texture features with patient survival, they discovered that entropy, homogeneity, and skewness predicted patient survival after treatment (28). Nakajo et al. extracted textural features from PET/CT scans of 52 patients with esophageal cancer receiving concurrent radiation. They concluded that texture-related characteristics could predict clinical response (29). The preceding study suggests that we can analyze the heterogeneous information of esophageal cancers based on the radiomics features of pretreatment CT and then develop a model to predict the efficacy of radiation in patients. In the present study, we discovered that the 90th percentile of the first-order statistical parameters may differentiate between responders and nonresponders.Texture features reflect the spatial distribution of pixels within the tumor (26), and the spatial distribution of pixels is more irregular in heterogeneous tumor pictures. The two-dimensional gray area size matrix’s large area dominance feature (glszm HighGrayLevelZoneEmphasis) indicates more related areas in the image, indicating a coarser texture, and treatment responses can be classed accordingly. Additionally, the Gabor wavelet transform was employed to extract additional features. As a short-time Fourier transform, Gabor wavelet transformations can deconstruct a picture into its component frequencies and directions (30). This study also demonstrates that Wavelet.Hll firstorder Skewness, Wavelet.Lhh glcm ClusterShade, Wavelet.Hll glcm ClusterShade, Wavelet.Hll firstorder Maximum, and Wavelet.Hhl glcm ClusterShade may discriminate the treatment response.

This study has some limitations. First, this study lacked multicenter validation and was conducted at a single institution. Nonetheless, the data in this study were obtained from a single CT scanner, which ensures equal scanning parameters and eliminates the influence of multiple devices and scanning parameters on picture characteristics. Second, a previous study showed that genes such as CXCR-2 and cyclin D1 are closely related with the prognosis of tumors (31). The incorporation of genetic characteristics into the radiomics model is vital.

## 5 Conclusion

In this study, a noninvasive, comprehensive, and individualized radiotherapy efficacy prediction model was developed by retrospectively analyzing the radiomics features of pre-radiotherapy CT images of patients with esophageal cancer. Validation and model evaluation were also performed. The model integrated radiomics features and clinical factors with good predictive accuracy, providing a cost-effective and simple evaluation technique for determining the effectiveness of radiation for esophageal cancer.

## Data availability statement

The original contributions presented in the study are included in the article/**Supplementary Material**. Further inquiries can be directed to the corresponding author.

## Ethics statement

The studies involving human participants were reviewed and approved by ethical committee of Mianyang Central Hospital. The patients/participants provided their written informed consent to participate in this study.

## Author contributions

XD, guarantor of integrity of the entire study and manuscript editing. JL and XY, study concepts and design. JL and XY, literature research. JL, XY, XM, TW, XZ, GF, TD, and XD, data collection. JL, XY, and XD, data analysis. JL and XY, manuscript preparation. All authors contributed to the article and approved the submitted version.

## Supplementary material

Supplementary Figure 1 Analysis flowchart. The enhanced computed tomography (CT) images **(A, D)**. GTV of manual segmentation **(B, E)**. Generation of 3D ROI **(C, F)**.
