# Supplementary figures and images for: Predicting the efficacy of radiotherapy for esophageal squamous cell carcinoma based on enhanced computed tomography radiomics and combined models
Source: Front Oncol. 2023 Mar 16;13:1089365. doi: 10.3389/fonc.2023.1089365 (PMC10061127; doi:10.3389/fonc.2023.1089365)

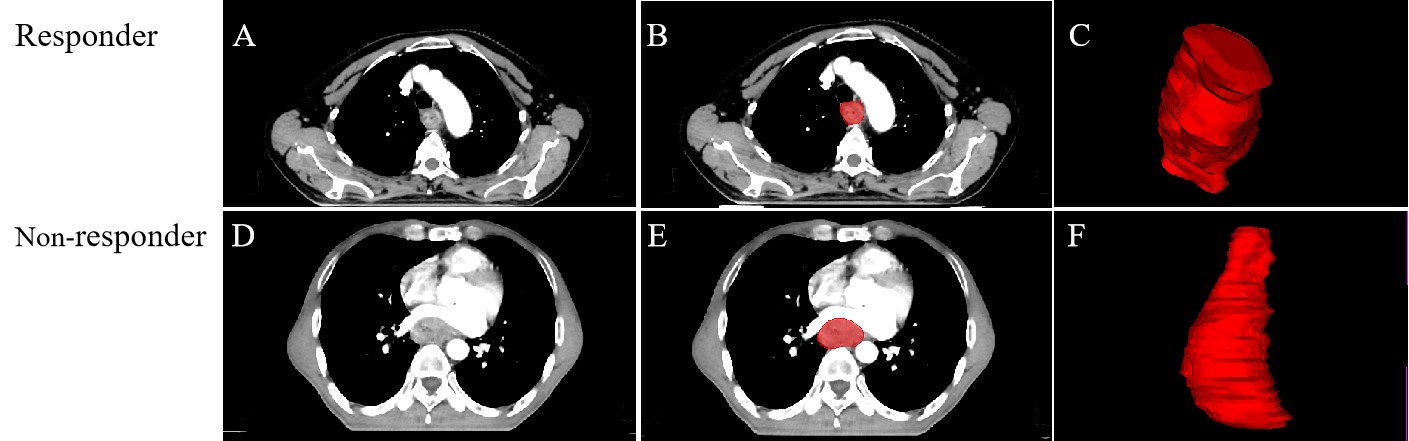

Supplement: Supplementary Figure 1 — Analysis flowchart. The enhanced computed tomography (CT) images (A, D). GTV of manual segmentation (B, E). Generation of 3D ROI (C, F). [file Image_1.jpeg]
