# Supplementary material for: Predicting the efficacy of radiotherapy for esophageal squamous cell carcinoma based on enhanced computed tomography radiomics and combined models
Source: Front Oncol. 2023 Mar 16;13:1089365. doi: 10.3389/fonc.2023.1089365 (PMC10061127; doi:10.3389/fonc.2023.1089365)
Supplement: Supplementary file 2 [file Table_1.docx]

Supplement Table1 Multivariate analysis of factors in training set

| Variables | OR(95%CI) | *p* |
| --- | --- | --- |
| Intercept | 0.102(0.018-0.457) | 0.005 |
| Gender | 2.028(0.703-5.945) | 0.189 |
| Thickness | 2.033(0.937-5.039) | 0.0979 |
| Rad-score | 15.326(4.069-71.952) | <0.001 |
